# Dentistry Versus Medicine from a Physician’s Viewpoint

**Published:** 1917-07

**Authors:** H. P. Williams


### DENTISTRY VERSUS MEDICINE FROM A PHYSICIAN'S VIEWPOINT

BY H. P. WILLIAMS, M.D.

It is not long since I looked on the dentist as a sort of necessary nuisance whose function in the community was nothing more than to apply cold steel and ''yank'' an offending tooth from the mouth of the fellow who has been

---

[4] Limitations of the White Bean as a Dietary Component, editorial The Journal A. M. A., June 2, 1917, p. 1627.

spending sleepless nights walking the floor and thinking of spooks and hobgoblins and using language hardly permissible in a Sunday-school class while his tooth was kicking like the proverbial bay steer. Now, my idea of a dentist has materially changed, and I am proud to say to you that to my mind he is one of the most useful, and should be one of the most respected citizens of any community, and fills, or should fill, a place in the community which can not be filled by any one else or any other profession.

There was a time when there was some antagonism between the dental and the medical professions, each accusing the other of encroaching on his clientele, but I think now that the dentist who feels thus is the fellow who never thinks of his own profession without hearing an ''Anvil Ring,'' and the medical doctor who feels thus is the fellow whose literature is the medical books of his grandfather and an occasional sample copy of the medical *World,* and who, when peering out through the cracks of his musty office, see a ''barber's pole'' in all its stately splendor. No, gentlemen, the time now is that with each member of both professions, who is in anywise progressive and is keeping abreast with the progress of the times, realizes that we are allied to each other, are copartners in our work, and almost daily in need of each other's council. You who stand as sentinels of the oral cavity too well know that species of all the disease-producing bacteria infest the mouth, there they propagate, multiply and flourish, are mixed with the food in mastication and sent through the throat to the stomach and bowels to ply their nefarious vocation in the alimentary tract. Or, from a focus of pyorrhea, they are ready at any moment to enter a lymphatic and be hurried away to a neighboring gland to produce a lymphangitis, or maybe be swept off in the blood stream to the remotest parts of the body, there to produce a general infection such as pyemia, rheumatism, or white swelling, you see how negligent would be the physician who fails to recognize the focus of infection, and how helpless he would be without the aid of a progressive dentist.

We, as physicians, in the whirlwind of our daily work, are too prone to carelessness in our examinations, and so, often fail to discover the real pathology of disease; we form habits, and habits once formed are apt to cling to us. We look into the mouth and see only the tongue and make an estimate of the covering which spreads over it. We lose sight of the important fact that there are other organs in the mouth; we do not see the large tonsils filled with crypts, or even think of the soft, spongy gums therein, or discover the pus exuding from beside the teeth, hence never suspect that here in this mouth swarm colonies of bacteria ready to be swept through this gateway into the bronchial tubes and into the stomach, there to enter the general system. Many are the times that bacteria from the mouth, on entering the stomach, come in contact with an irritated surface and find lodgment in the gastric mucosa with the inevitable result of a local infection with nausea and vomiting, "our classical congestion," of the stomach, or further down the tract they find lodgment, with the result of infection there, and though we apply remedies supposed to be specifics for this trouble, no improvement follows, or if any improvement it is only temporary and relapse soon follows, and why, we ask ourselves? If we had found the etiology, the answer is easy. Back up in the mouth the well-springs of bacteria from whence we had the original infection, the same bacteria are being poured out in innumerable hosts and floating down the throat to reinforce its fellows in the stomach and bowels, and thus perpetuate the pathology there. This process reminds me of a young woman in my care once, who was about to undergo an operation. Just before she was given the anesthetic she called her husband to her and said: "My dear, if I have done anything to vex or offend you, and I know I have, I want you to forgive me," and in the next breath she added, "But if I come out of this all right I'll do it again, *I'll* promise you." So with these cases of mouth infection: they may get apparently well, but they will either relapse or have some other form of infection.

Sickness and death strike terror to the hearts of all men everywhere. Nature's strongest instinct is life preservation. This instinct pervades all forms of life. To it is due the perpetuation of life. We call the antithesis of health, disease. The guardian of health and the relentless foe of disease is the physician. Let me pause here to say, lest I be misunderstood, that I include under the term physician all who labor in this field—let him be an internist, a dentist, a surgeon or the family doctor. If, then, it is given to us to foster, protect and guard the health, the thing of supreme value to all classes of people, should we not be proud of our calling, and see that such pride is not unmixed with a due sense of the grave responsibility which we assumed to our fellow man upon donning the mantle of the greatest of all professions?

We are now living in a day of specialism, and there is no greater tendency in any profession to specialize than in our profession, and what is this amounting to in a general way? The answer is easy: the people are getting better, are getting more scientific service. They are getting service which is reflected in the results, results they never got before and such as would until today be impossible for them to secure had the profeession not realized and acted on the realization that by sectionizing the human body, then selecting a small portion and making a real study, and a thorough preparation to treat only this limited part, that they could render more general satisfaction.

The dentist chose the mouth for his field of endeavor. Just why, I do not know. Maybe you can explain. However that may be, we are all aware of one thing, and that is the mouth is prolific with pus-producing bacteria, and from their ravages comes the most of the pathology encountered there. It was said by one noted writer that the crusts of tartar found on the teeth are only so many tombstones erected to the departed germs. Whether that be true it matters not, yet it remains the same, the germs are there and there to stay, and fortunate is the dentist who does not sooner or later encounter them in such quantities and of such virulent type that he is not put to his wit's end to control them. The dentist usually meets this formidable foe in its own camp; that is, you deal with the nidus or focus of infection, but the unlucky surgeon meets him after he is metastasized, and in place of a small nidus of infection we have a lymphatic gland or a chain of lymphatic glands, or multiple chains; we have them swept by the blood stream to the pericardium or the myocardium, to the valves of the heart, to the joints, to the marrow of bones, to the brain, in fact to all portions of the body except perhaps the hair and nails. So you see how our troubles multiply when we come to metastatic infection. And I think you will readily see how badly the surgeon needs the assistance of the dentist when he comes in contact with these infections, as the camp of the enemy is in the mouth, and from its stronghold there it is shelling the entire body, sending out reinforcements on every side the surgeon makes an attack. The dentist hurls a bomb into the camp by breaking up and destroying the focus of infection, when the surgeon has but little to do to win the battle.

The medical profession in general is delighted to pay tribute to the dental pathologist for his pioneer discoveries and invaluable contributions to the isolation and descriptions of the bacteria infecting the mouth, and I am quite sure no dentist of the present day but pays homage to them for these discoveries and teachings, for at this pivotal point dentistry, alike with all other branches of medicine, began to make rapid strides forward. Prior to this the dentist, whose prowess lay in the celerity with which he could separate one from his ivories, and whose sole and highest ambition was to be known as the lightning tooth puller, has so changed that now he seeks to excel in prophylaxis, and finds his chief delight in preserving to us that which his predecessor could only extract.

All intelligent, reading, thinking people know that most of the sickness we have is caused by living bacteria and are clamoring for knowledge in the lines of sanitation and prophylaxis. The whole trend of scientific endeavor is toward prevention rather than cure of disease.

A gigantic wave of instruction to the laity, such as the world has never seen or known, is sweeping over the country, teaching the people how to keep well. Columns are constantly appearing in the great dailies and weeklies of the country, and these silent monitors do much in the homes to diffuse the knowledge of keeping well. What part are we playing in this game? Do you play your part in this game? This is for you to answer. Each and every dentist, every internist and every surgeon should be teachers of the community in which he lives. The dentist, as a specialist of the oral cavity, the beginning of the great *prima via*, is especially fitted by his training and by his associations with his patrons; is in the most favorable position to teach sanitation, to teach oral hygiene, to teach oral prophylaxis, and he should not fail to improve every opportunity to do so. He should teach in his office, in his chair, in public, and especially should he teach in the schools of the country whenever an opportunity presents, and in this way mold into the young minds truths that will live when you and I are no more. The foundation of the great fundamental truths of prophylaxis being thus laid by the dentist, the superstructure can be carried on by the conscientious physician to completion and will stand a living monument to our professions in years to come.

Do not get the idea that I would decry an ample income to the dentist as a pecuniary reward for his services; be that far from me, for the laborer is worthy of his hire. And by way of parenthesis, I know of no way by which you can better advertise your knowledge and ability and build up an appreciative clientele than along the lines I have suggested.

I might ask you, are you making a success in your chosen profession? If so, what is your success? Are you using your profession as a bread and butter proposition? Are you measuring your success by the dollars you get out of it? If so, you are in the wrong place here tonight. It will be an utter impossibility for this Association to help you. You have been slipping back, and slipping back every day since your advent into the fraternity, and you are so far back that all the powers that be can not redeem you; but on the other hand, if you are in love with your profession, if it gladdens your very heart to relieve suffering, if it delights you to do good and be of service to your fellow men, then you can be numbered among the progressive dentists of your state, and measure your success by a large, appreciative, cash-paying clientele, composed of all the good citizens of your community.—*Western Dental Journal.*

